# Supplementary material for: Assembly and the gating mechanism of the Pel exopolysaccharide export complex PelBC of Pseudomonas aeruginosa
Source: Nat Commun. 2025 Jun 5;16:5249. doi: 10.1038/s41467-025-60605-8 (PMC12141448; doi:10.1038/s41467-025-60605-8)
Supplement: Supplementary file 1 — Supplementary Information [file 41467_2025_60605_MOESM1_ESM.pdf]

Supplemental Table 1. Cryo-EM data collection, refinement and validation statistics

	PelBC (EMDB-51916) (PDB 9H80)
**Data collection and processing**	
Magnification	165,000
Voltage (kV)	300
Electron exposure (e–/Å^2)	60
Defocus range (μm)	0.5-3.0
Pixel size (Å)	0.727
Symmetry imposed	C1
Initial particle images (no.)	2,345,182
Final particle images (no.)	124,181
Map resolution (Å)	2,52
FSC threshold (0.143)	
Map resolution range (Å)	2.1-3.5
**Refinement**	
Initial model used (PDB code)	AlphaFold2
Model resolution (Å)	2.5
FSC threshold (0.500)	
Model composition	
Non-hydrogen atoms	35251
Protein residues	2252
Ligands	27 PTY
B factors (mean) (Å^2)	
Protein	61.19
Ligand	61.52
R.m.s. deviations	
Bond lengths (Å)	0.003
Bond angles (°)	0.675
Validation	
MolProbity score	1.14
Clashscore	2.50
Poor rotamers (%)	0.85
Ramachandran plot	
Favored (%)	97.47
Allowed (%)	2.53
Disallowed (%)	0.00

Supplemental Table 2. Sequences of used primers and synthesized genes

Name	Sequence (5'->3')
PelB-PlugI_Fw	ATGCGCGACAGCCTACGGCTCGG
PelB-PlugI_Rv	GCCGGTCTCGTCGGTCTCGCG
PelB-PlugO_Fw	GATGGCGCGCGCAGCCAGGAC
PelB-PlugO_Rv	CACGCGGTTCTCCAGGCGATTGCG
PelB-PlugS_Fw	GGCGCCGGCGGCACGCTC
PelB-PlugS_Rv	GGGAGCCGACTGGTAGCCGAAG
PelB-Δ876_Fw	AAAAAAGCTAGCAATGGCGAACGCACTCCCCAGGGCC
PelB-Δ876_Rv	TTTTTTGGTACCTCAGCGGCCGAAGCGCGTGCTG
PelC-W149A_Fw	GCGCGCCGGCgcgTCCCGGGAAA
PelC-W149A_Rv	GCCCCGCTGGTGCTC
LPP-SP_PelC (synthesized gene)	AAAAAAACCA TGGGCAAAGC TACTAAACTG GTACTGGGCG CGGTAATCCT GGGTTCTACT CTGCTGGCGG GCTGCTCCAG CTTCACCAGC GAAAGCGCTA CGCCGCTGGC GCGCGGCGCG CAGTGGGGCC TGGTGCCGCT GCTCAATTAT TCCCAGGCGC CGCAGGCCGG CGAGCGTGCC GAGCAGATCC TGCTCAGCGT GCTGGCCGAG GAGGGTGTGC GGCCGCAAAA AAA
pectate-lyase-SP_His-tag_PelB (synthesized gene)	CATATGAAATACCTGCTGCCGACCGCTGCTGCTGGTCTGCTGCTCCTCGCTGCCCAGCCG GCGATGGCCATGCACCATCACCATCACCATCACCATGCTAGCAATGGCGAGCCGGCGAT GCTGCAGCTGTGGTTCGAGCAGTTCCTCGACCAGCTCGCCGCCACCAACCAGGAGCCGC TGAAGGATAACTGGCTGGCCTGGGCGCGCGGCCGGGGCCTGAAGATCGGGCGCAACG AAGAGATCCAGGCCGCCCTGCGCAGCCAGAATCGCGCCGCCCTGCAACGCCTGCTGGA GCGCGGCGAGCTGGATCCGGCGCAGCGGGTCGAGGCGCTGGTGCGTCTCGGCCACGG CGGCGAAGCGCTGGGCGAGGCCCTGGGCGCACTGGGCGACGGCCACTCGCGCGACAA CCGCGAGCAGCTGCGGCGGCAGGCGGCGGAAATCCTCGAACGCACTCCCCAGGGCCTG CAACTGGGTTGGAACAAGCGCGACTTCGGCGGTCTCGACTTCAAGGGGCCGACGCTGC GCGCGGCGCGCCACCTCGGCGACGACTGGTACGCCGATCTCGAACTGGGCAGTGGCCG CTATCACGGCGATGCGCTGGATAGCTCGCTCCTCGGTAGCGAACGCAACGCCAGGCTGA CGTTGCGGCGCGAACTGGCCGATGGCTTCGCCGCGGCGACGCTCGATGGCAGCTGGCG CGACGACGAGGACCGCCACGGTCTGGGGGTGCTGCGCAATTGGCGGCTGAGCTCGCGC GACGAACTGGAAGCGGGCCTCGACTGGCACCGCGAGACCGACGAGACCGGCCTGATGC GAGCCCTCGGCATGCGCGACAGCCTACGGCTCGGCGGCCGCCACACCCTCAGCGGGCG CGACCAGTTGAGCTGGTCGCTGGCGCACAACCGCTTCTCTACCCGCCAGGGCGACGACC TCGGCAACGGCGAGGCGCTGTCCCTGGAATGGGCCCACACACTGTTCTTCGACGGCCCG GCCTGGCAACTGCGCGGCGGCATCGACTACCAGCGCAATCGCCTGGAGAACCGCGTGC CGGACGACCTGCTGGCGGCCCACGGCGGTGCGCTGGCGCTCGATGGCGCGCGCAGCCA GGACCTGCTGCAGGACCGCTATGGCCAGGTCTACCTCGGCAGCACCTGGCGCCGTGGCT TCCCTGGCGCGCTGAACCGCAGCCGTCCGCAATACACCTGGATCGTCGACACCCTGGCC GGCTGGCAGTGGACAGAGAAGGAGTTCAACTACGGCATCGATCTCGGCATCGGCATGG AGCTGCTGGGCGACGACGAACTGGCGTTCACCTTCGGCTACCAGTCGGCTCCCCAGGGC GGTGGCGGCGATGCCGGCGGCACGCTCGGGGTGACCTACAGCACGCGCTTCGGCCGCT GAGGTACC

Supplemental figures

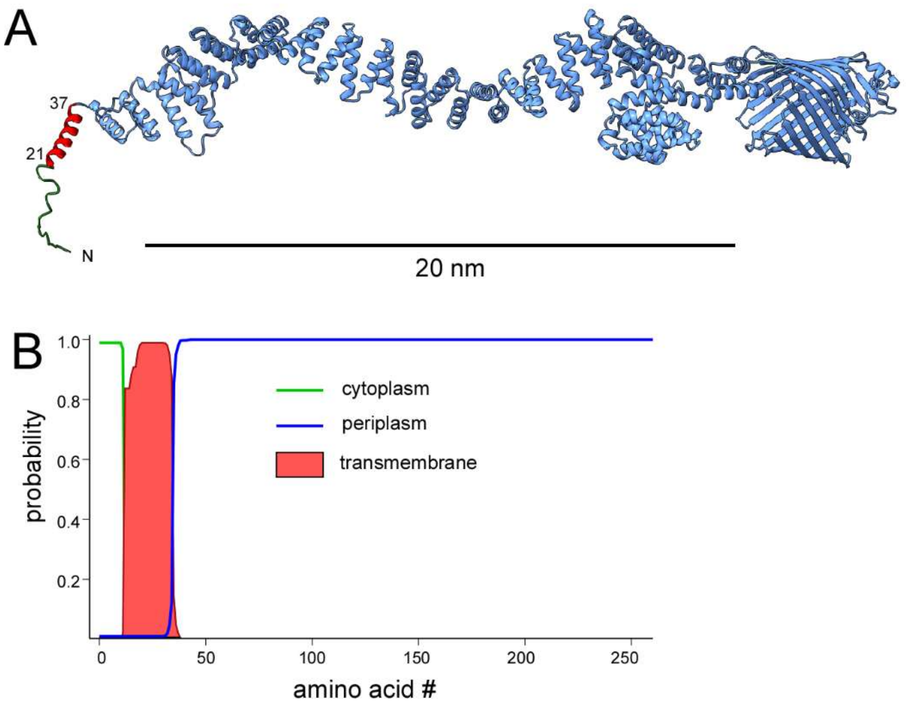

Supplemental Figure 1. Outer membrane protein PelB is anchored in the inner membrane.

(**A**) AlphaFold 3 model of *P. aeruginosa* PelB predicts an α-helix formed at the N-terminal end of the protein (shown in red) prior the periplasmic TPR domains.

(**B**) TMHMM algorithm (https://services.healthtech.dtu.dk/services/TMHMM-2.0/) predicts a hydrophobic transmembrane helical domain within the same region of PelB, residues 21-38 (plot for the first 260 aa is shown). No signal peptide is identified using SignalP 6.0 service (https://services.healthtech.dtu.dk/services/SignalP-6.0/).

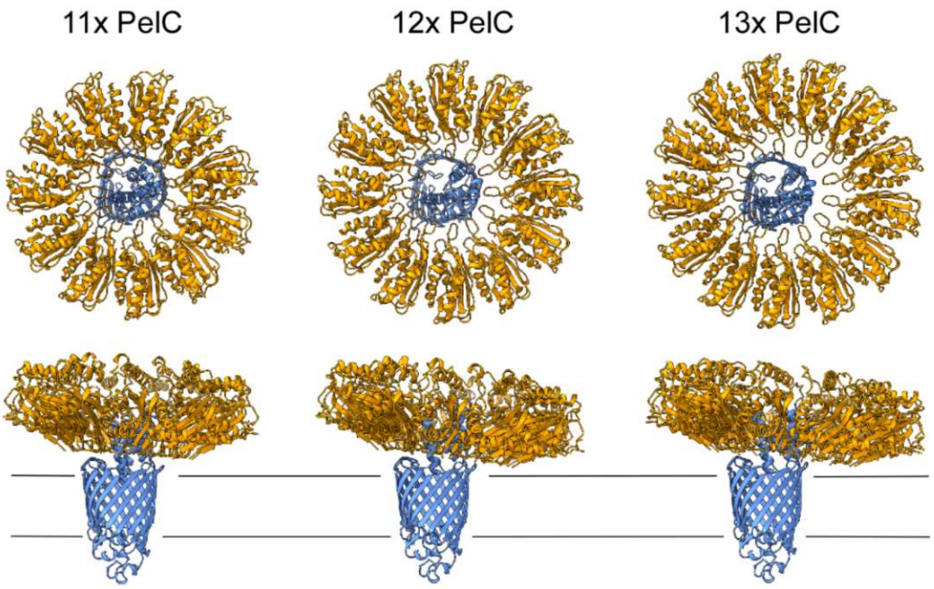

Supplemental Figure 2. AlphaFold3 models of the PelBC complex assuming 11, 12 and 13 PelC subunits (shown in gold) bound to a single PelB β-barrel (shown in blue).

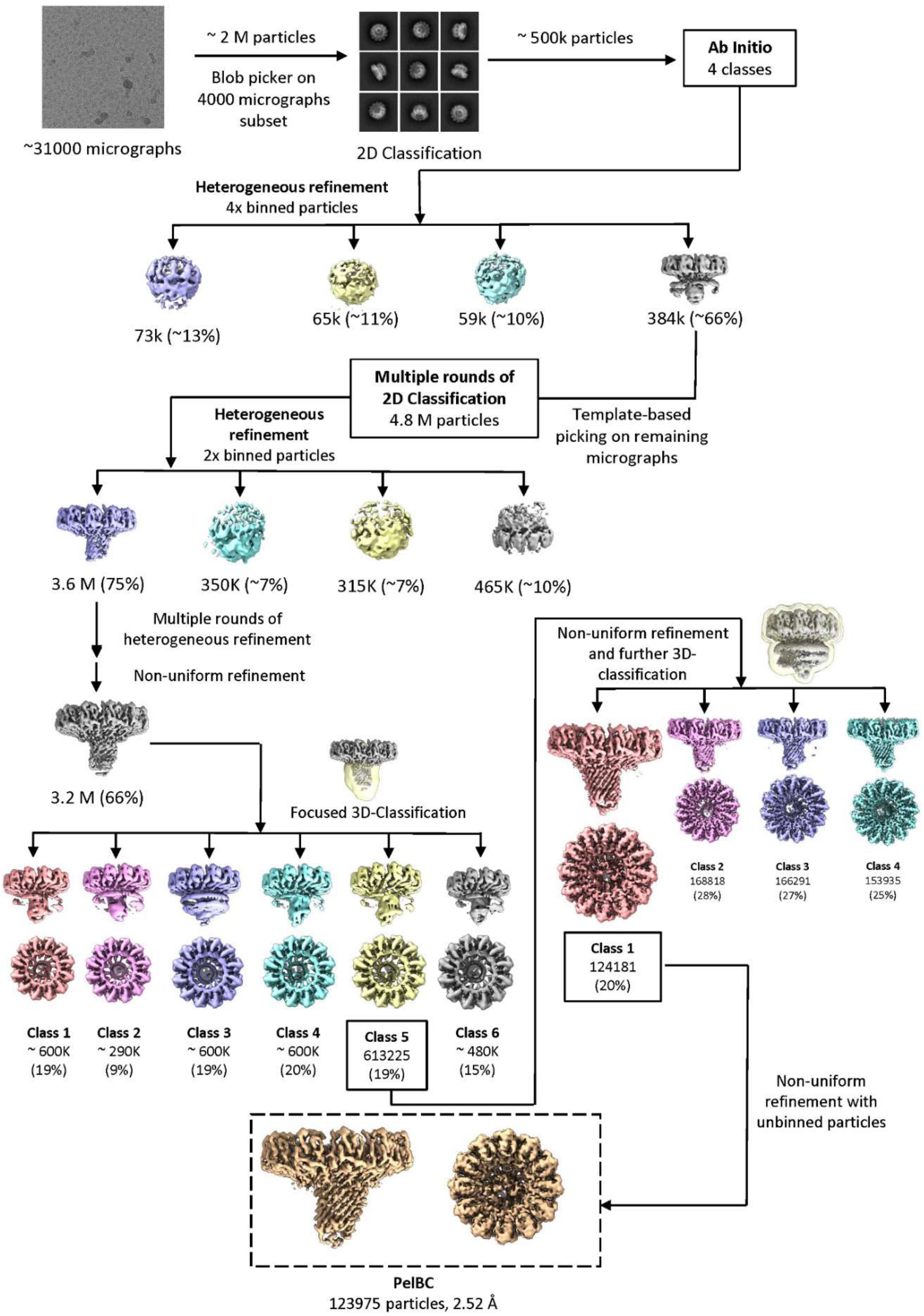

Supplemental Figure 3. Single-particle analysis towards the PelBC structure. Summary of the sorting/refinement procedures performed upon single-particle analysis of the cryo-EM data set of the nanodisc-reconstituted PelBC complex.

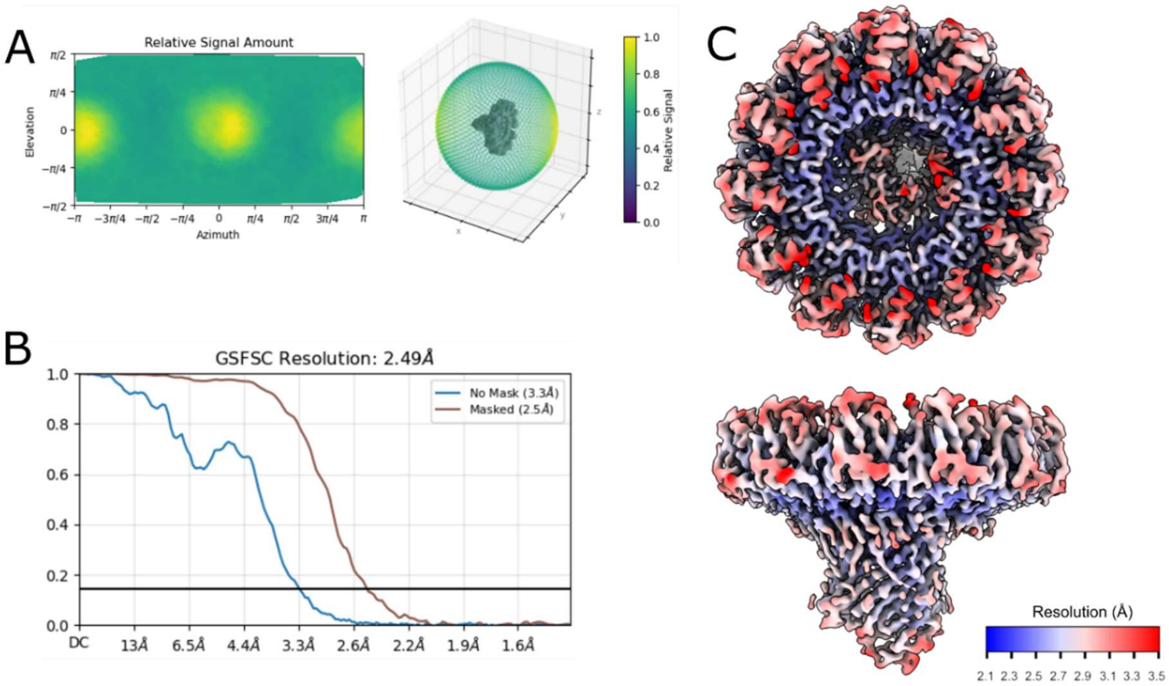

Supplemental Figure 4. Global and local resolution of the PelBC map.

(**A**) Angular distribution plot for the final reconstruction obtained from cryoSPARC.

(**B**) Gold-standard Fourier shell correlation resolution curve for the final PelBC map, displaying resolution with a mask automatically generated by cryoSPARC.

(**C**) The final PelBC map colored according to the local resolution (determined by cryoSPARC).

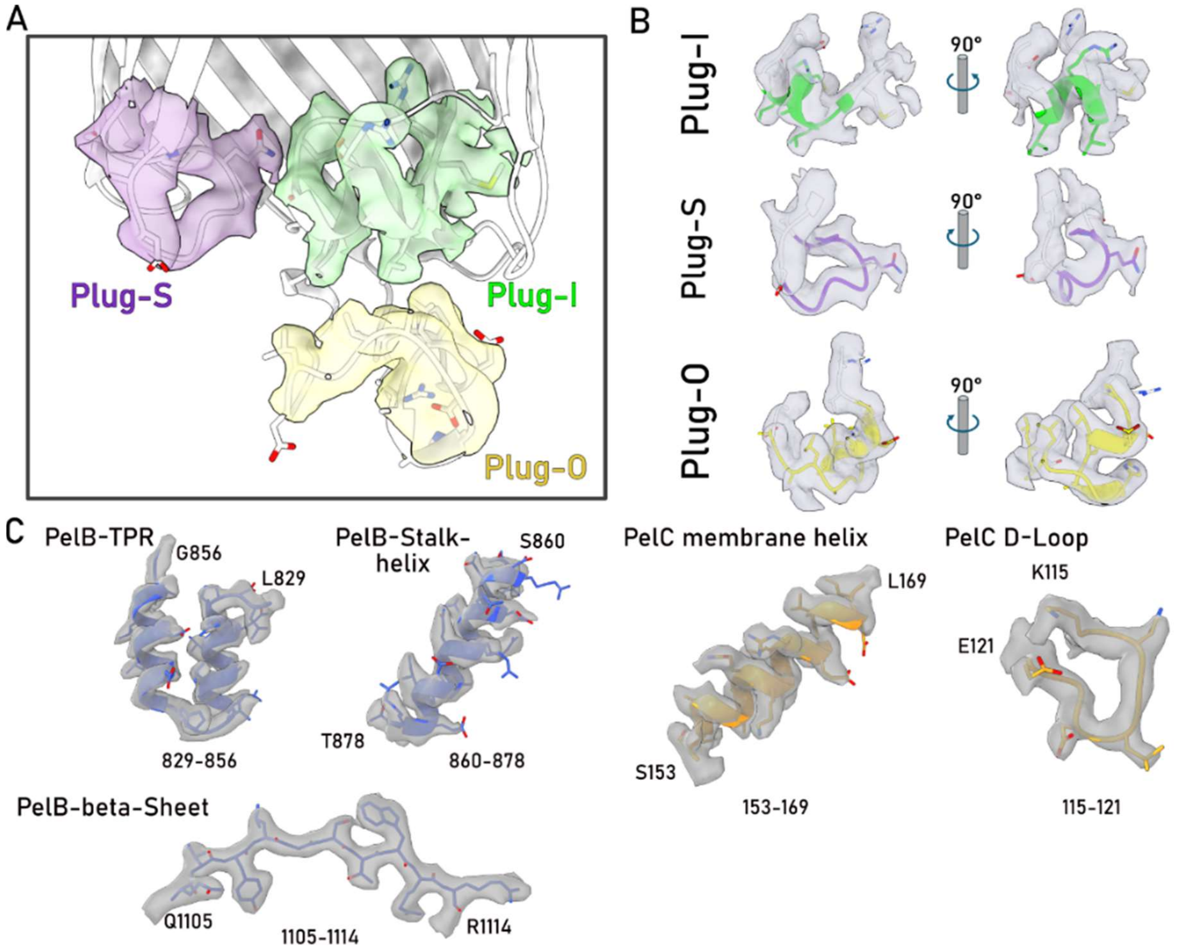

Supplemental Figure 5. Modelling the structural elements of the PelBC complex.

(A) Zoom-in to the extracellular loops/plug domains of PelB. The fragments of the cryo-EM density map are overlaid with the model of the protein.

(B) The extracted densities of the plug domains shown in different projections.

(C) Selection of PelBC structural elements with the corresponding densities from the cryo-EM reconstruction.

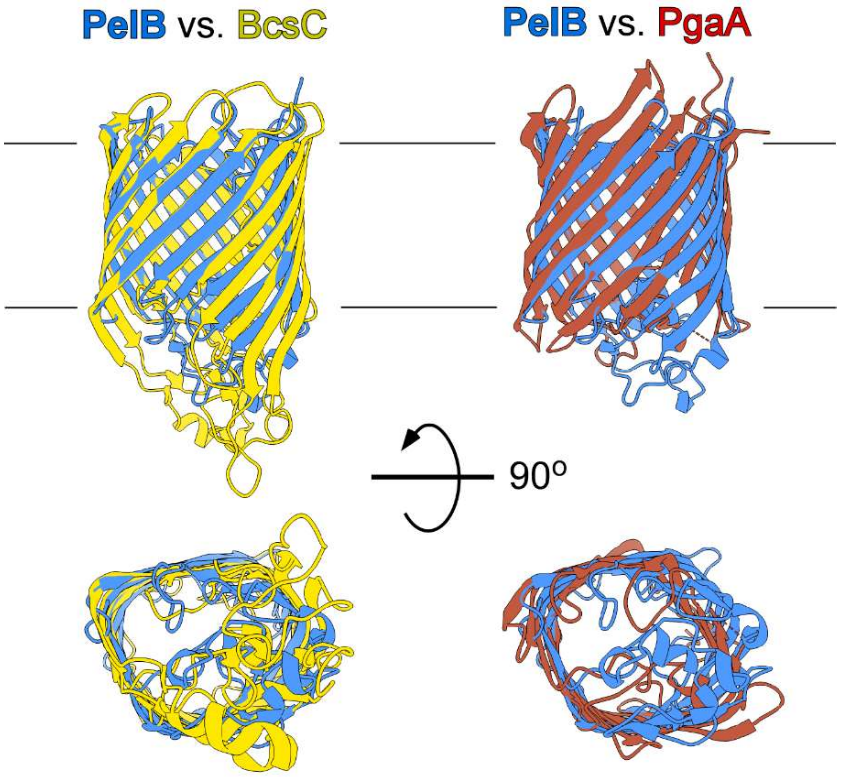

Supplemental Figure 6. Conserved architecture of the bacterial outer membrane transporters of exopolysaccharides. Structure of *P. aeruginosa* PelB β-barrel superimposed with *E. coli* BcsC (PDB ID 6TZK) and *E. coli* PgaA (PDB ID 4Y25), views in the membrane plane and from the extracellular side.

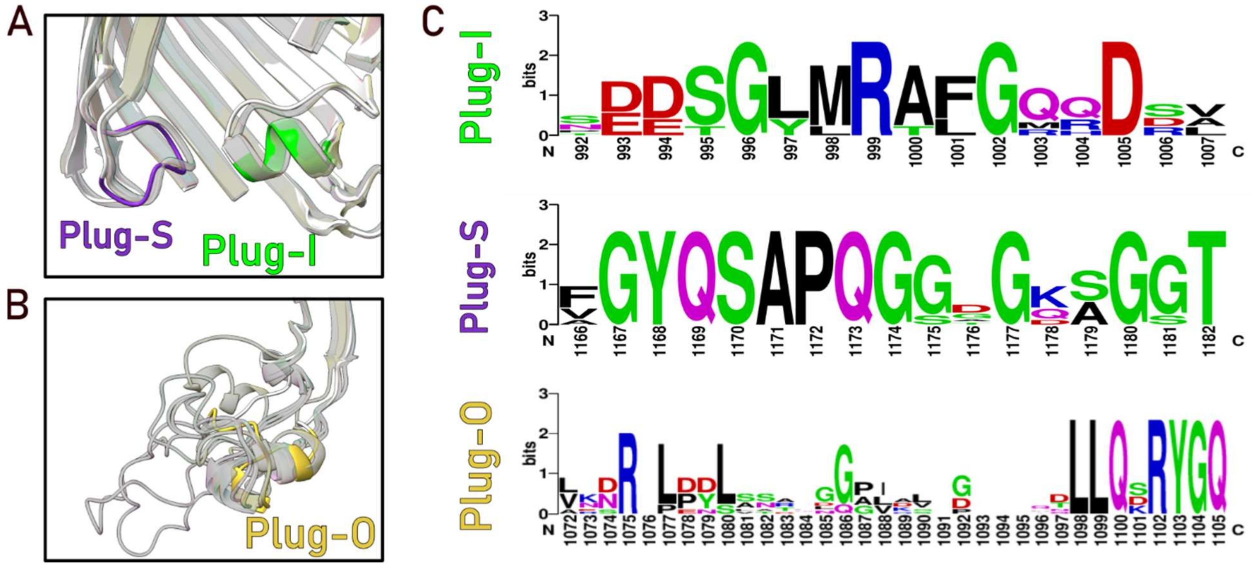

Supplemental Figure 7. Conservation of the plug domains among *Pseudomonas* species. The structures (**A, B**) and the sequence logo (**C**) are based on PelB of *P. aeruginosa* PAO1*, P. mandelii, P. oryzihabitans, P. protegens, P. simiae, P. sp.,* and *P. trivialis*.

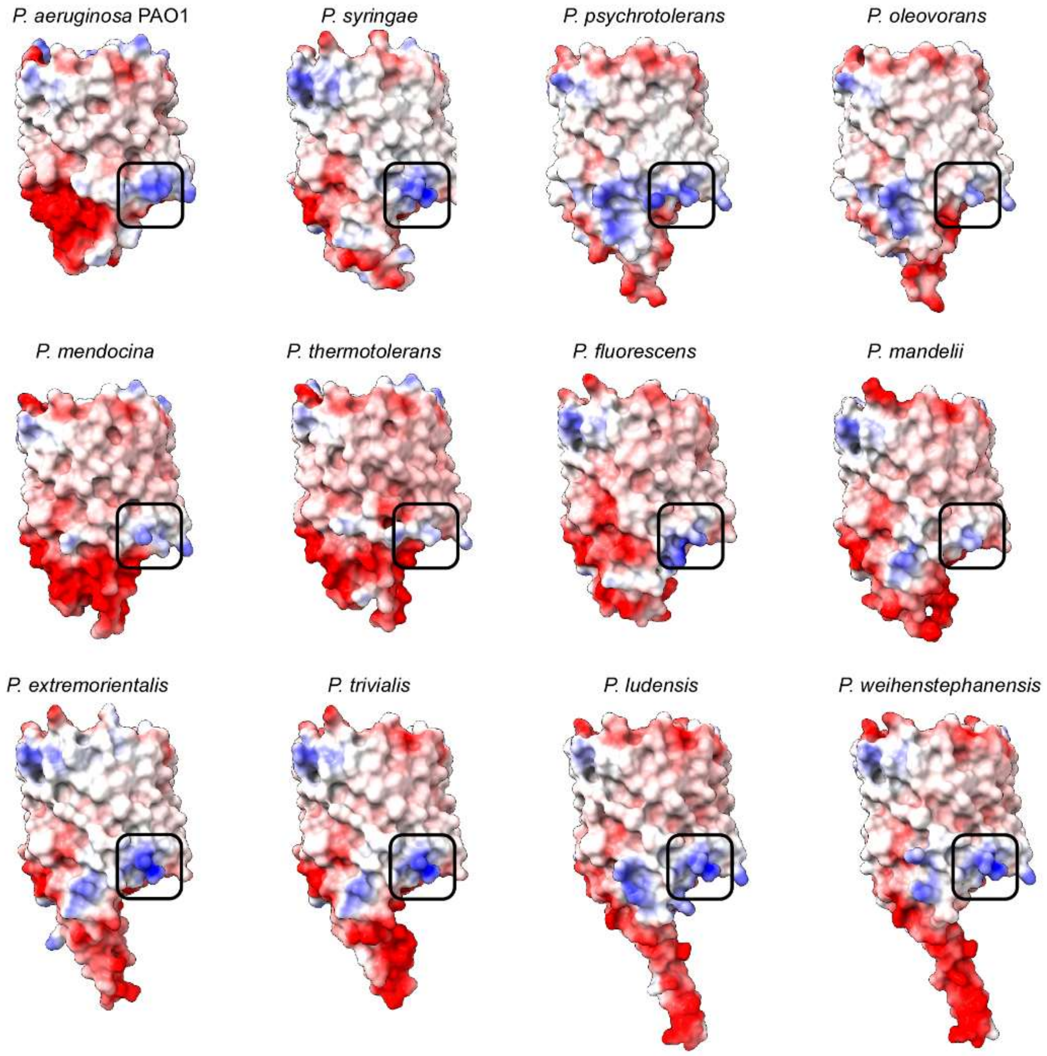

Supplemental Figure 8. AlphaFold models of PelB homologs from the indicated *Pseudomonas* species highlight the conservation of the cationic site Arg-1071/Arg-1102 at the extracellular side of the β-barrel (black square) and the diversity of the exposed Plug-O domains.

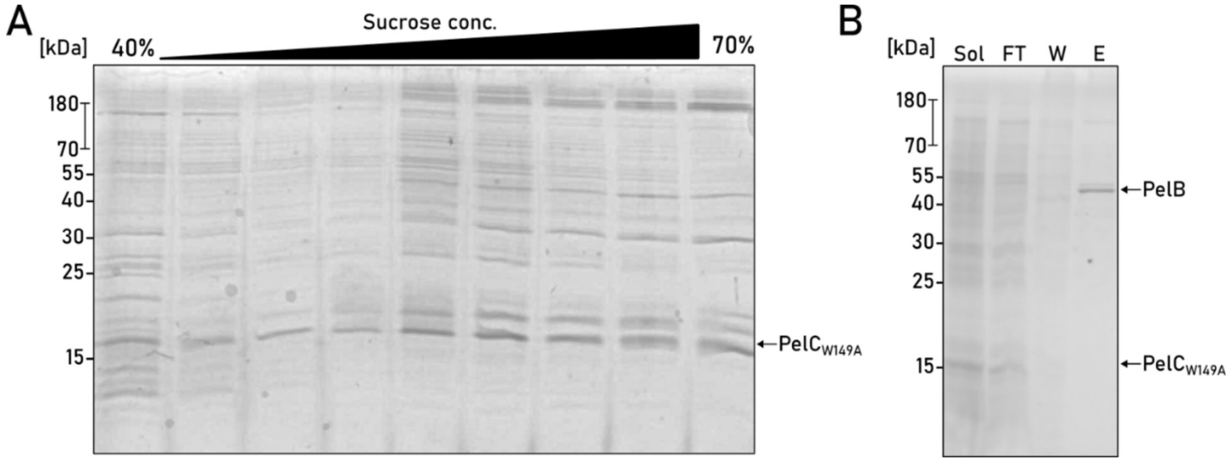

Supplemental Figure 9. PelC$_{W149A}$ is targeted to the outer membrane but does not form stable complex with PelB.

(**A**) SDS-PAGE of the sucrose density gradient shows localization of the PelC$_{W149A}$ mutant in the late high-density fractions corresponding to the outer membrane vesicles.

(**B**) SDS-PAGE of IMAC purification of co-expressed PelB and PelC$_{W149A}$ shows that the mutated PelC$_{W149A}$ subunits are not co-purified with the His-tagged PelB. Loaded fractions: "Sol" - detergent-solubilized material; "FT" - flow-through; "W" - wash, "E" - elution.

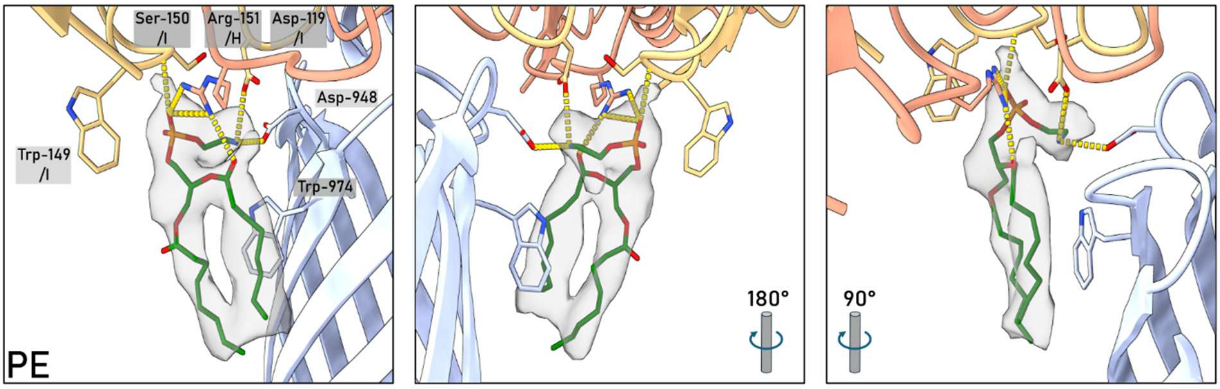

Supplemental Figure 10. Phosphatidylethanolamine molecule modelled at the PelBC interface. Different views illustrate docking of a PE lipid within the resolved density. Potential hydrogen bonds with PelB and PelC subunits are indicated as dashed yellow lines.

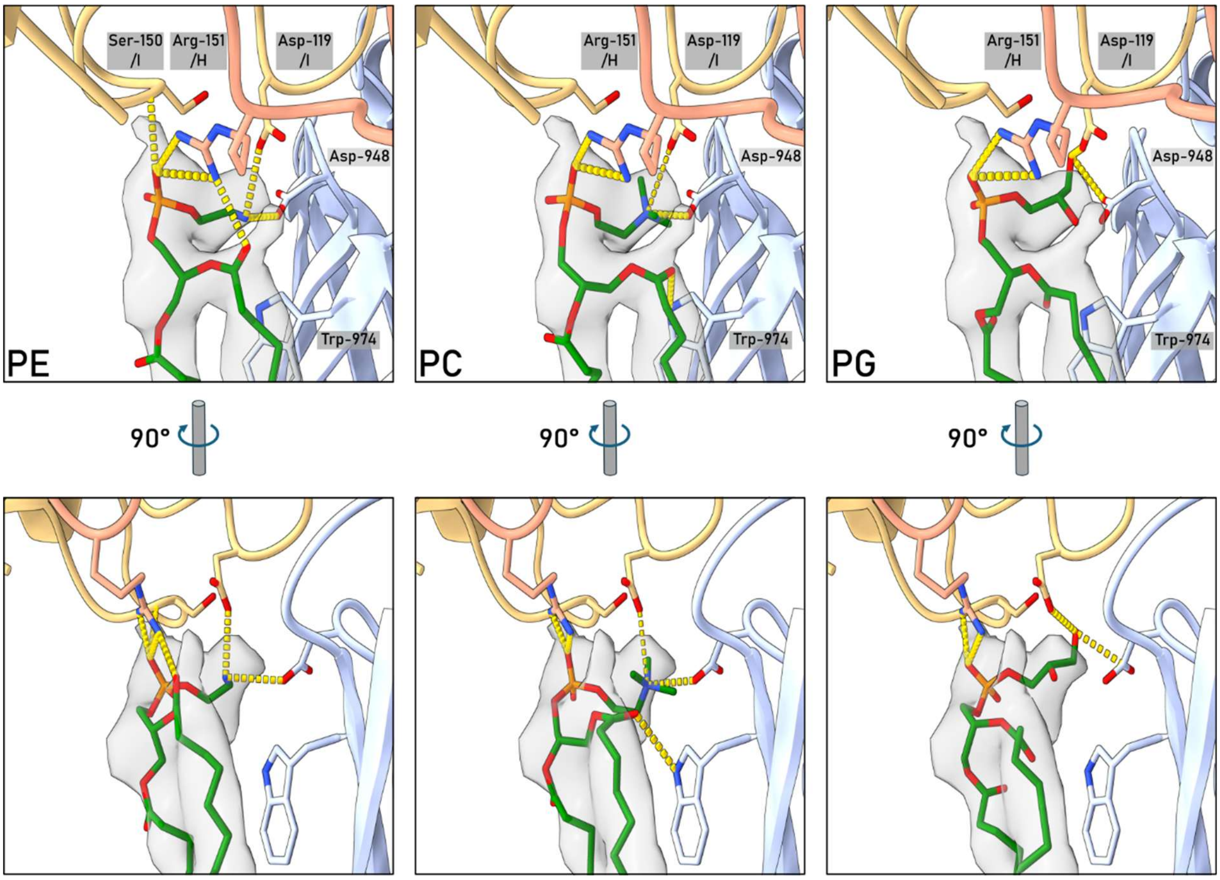

Supplemental Figure 11. Modelling a non-annular lipid at the PelBC interface. Phosphatidylethanolamine (PE) may form the most extensive interaction network with PelBC complex and ensure the optimal fit into the observed lipid density as compared to phosphatidylcholine (PC) and phosphatidylglycerol (PG).

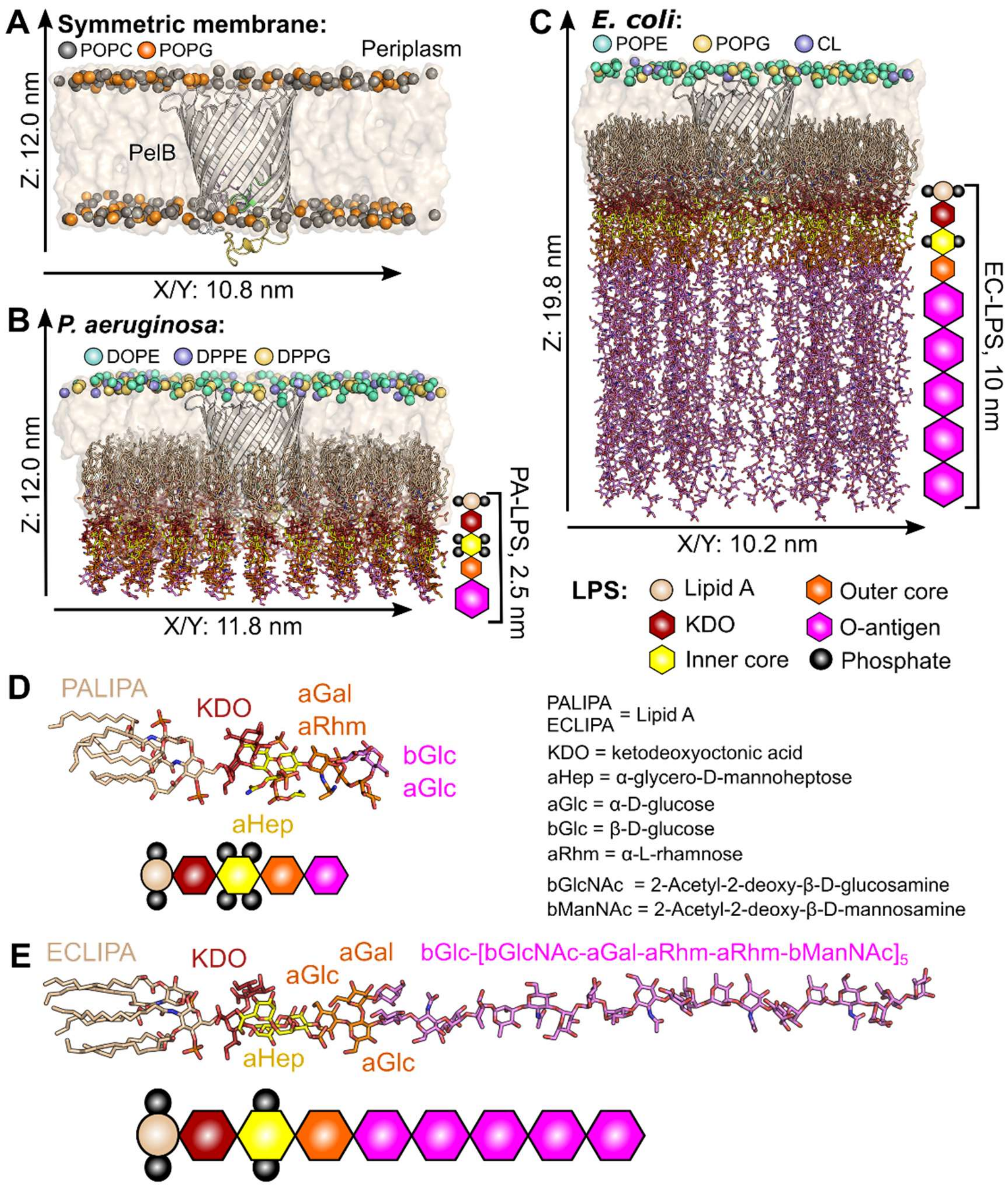

Supplemental Figure 12. The PelB β-barrel systems for the MD simulations. (**A**) The POPC:POPG system reflects the composition of PelBC nanodiscs used for the structure determination with 150 mM NaCl or additionally with 100 mM Ca^{2+} ions.

(**B, C**) CHARMM-GUI web server was used to model native-like membranes with LPS of *P. aeruginosa* (PA; inner leaflet DOPE:DOPG:DPPE) and *E. coli* (EC; inner leaflet POPE:POPG:CL) at 150 mM KCl and 150 mM NaCl as ion concentration, respectively.

(**D**) The structure of the used LPS for *P. aeruginosa* (PA) and **(E)** *E. coli* (EC) are depicted**.** In general, the LPS consists of the lipid A (wheat), ketodeoxyoctonic acid (KDO; dark red), the inner (yellow) and outer core (orange), as well as the repeated O-antigen units, where its length can be different for each organism. Phosphate groups are depicted as black circles at the sugars.

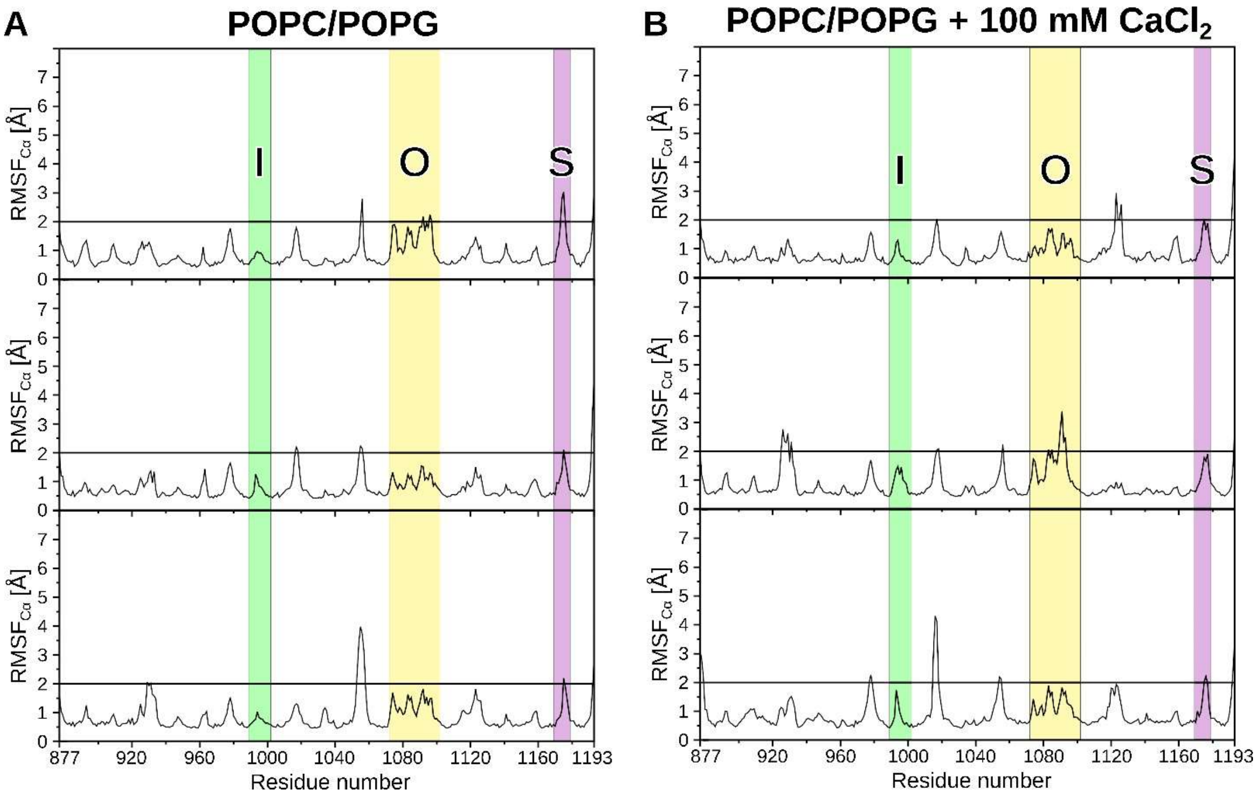

Supplemental Figure 13. Dynamics of the PelB β-barrel in the POPC/POPG membrane system simulated in triplicates at 25°C and 150 mM NaCl without (A) and with 100 mM $CaCl_2$ (B). To determine the flexibility, we used the root-mean square fluctuation (RMSF) of the Cα atoms per residue over the time, where residues over 2 Å are considered as flexible over 500 ns. The regions corresponding to Plug-I, Plug-O and Plug-S loops are highlighted.

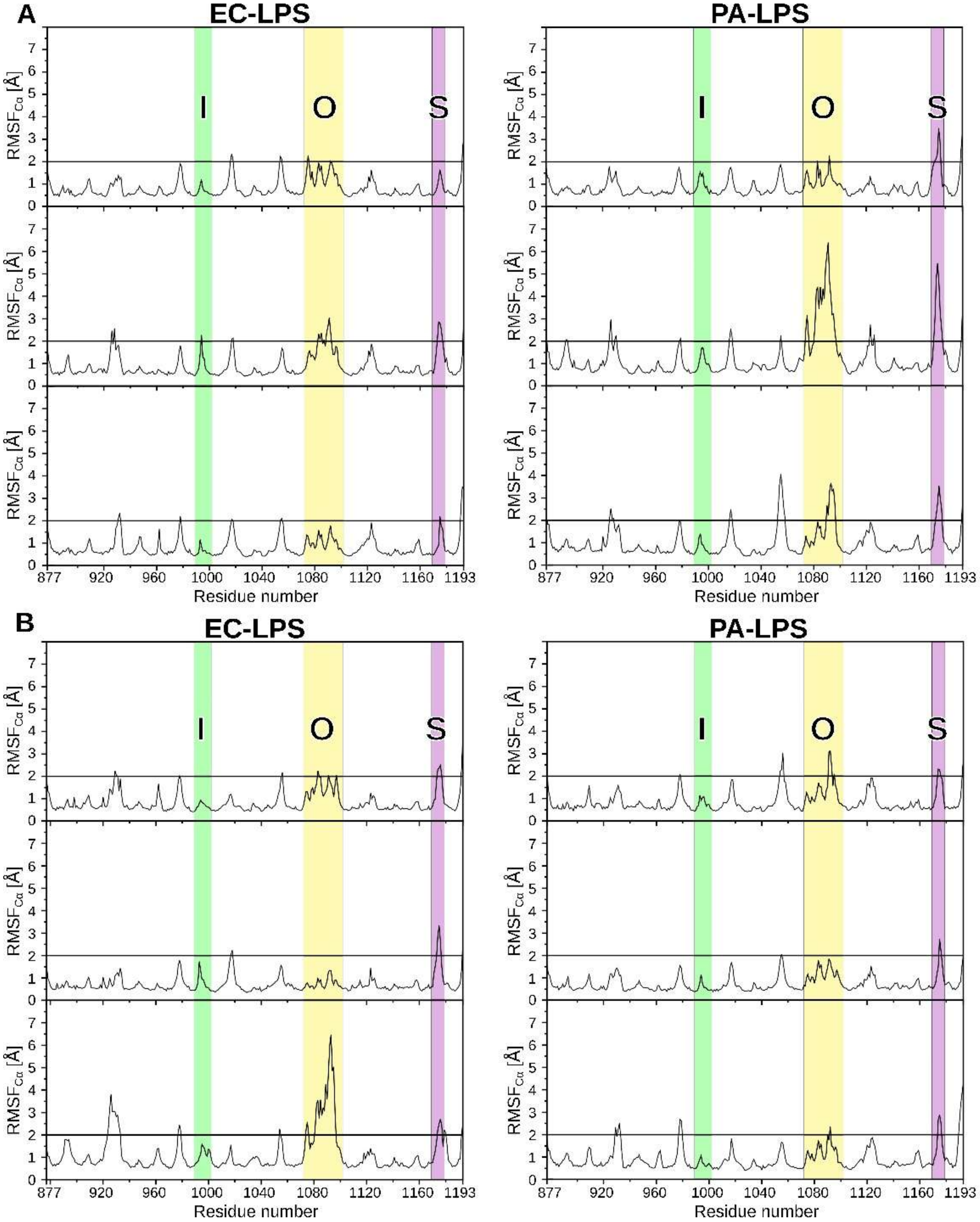

Supplemental Figure 14. Dynamics of the Plug loops in the native-like LPS-containing membranes. Modelled conditions are **(A)** 37°C and 150 mM KCl, and **(B)** 25°C and 150 mM NaCl. The regions corresponding to Plug-I, Plug-O and Plug-S loops are highlighted.

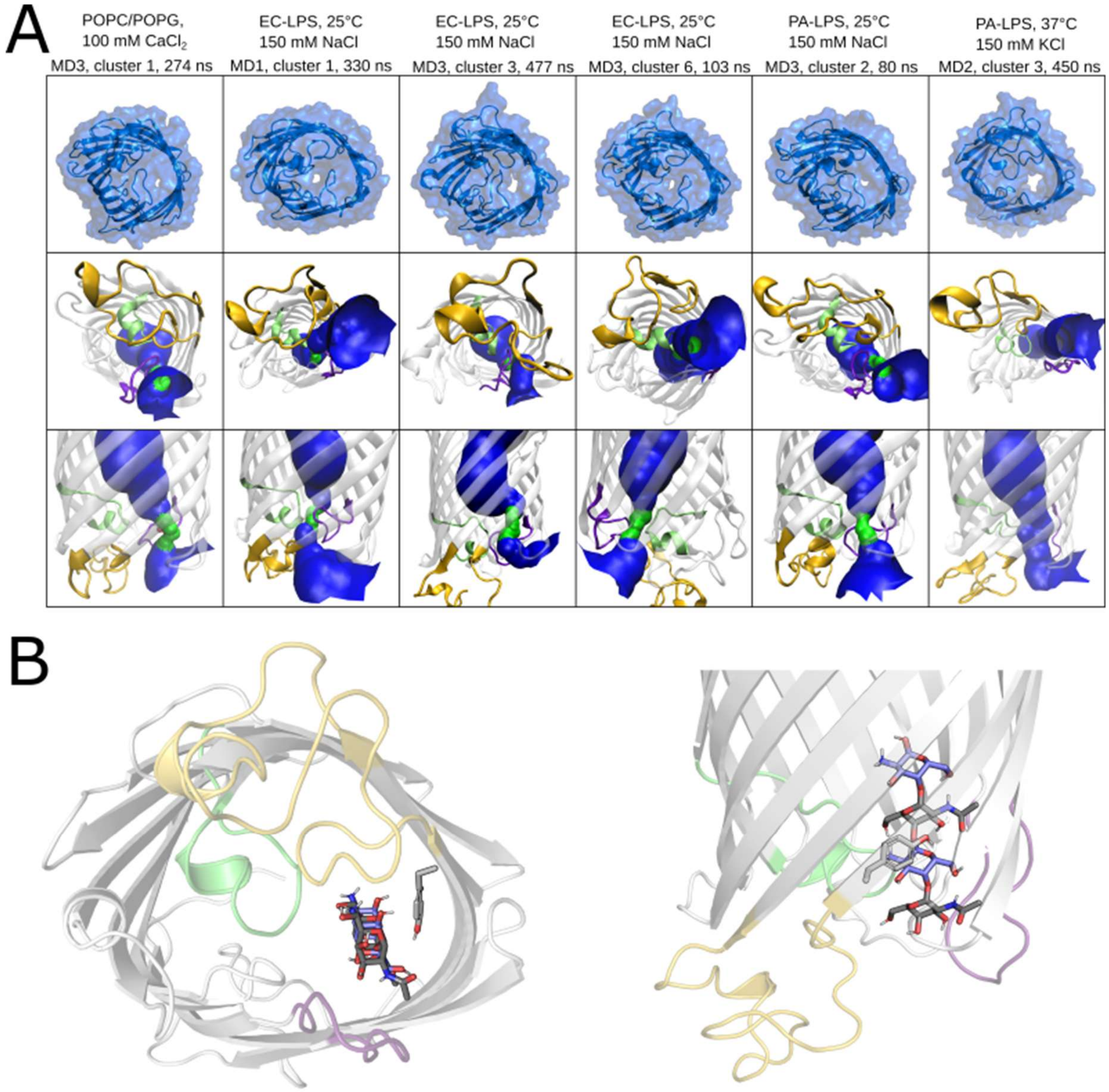

Supplemental Figure 15. The PelB β-barrel acquires an ensemble of transient open conformations. (**A**) Conformations sampled by PelB barrel upon the MD simulations in different membrane systems reveal narrow tunnels formed between the extracellular loops. The tunnels too narrow for passage of water molecules are colored in red, the tunnels sufficiently wide for a single water molecule are in green, and those accessible for multiple water molecules are in blue. The tunnel analysis was done with HOLE algorithm and Python package MDanalysis.

(**B**) A mimetic of the Pel exopolysaccharide, GalNAc-GalN-GalNAc-GalN, can be docked within the pore-forming conformation of PelB (PA-LPS at 37 ^{o}C). The conserved Tyr-1103 of PelB is shown in grey.

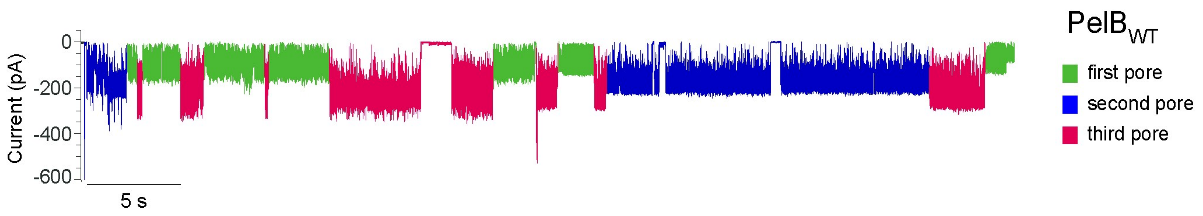

Supplemental Figure 16. A representative ion current profile recorded in presence of multiple wild-type PelB molecules incorporated into the lipid bilayer.

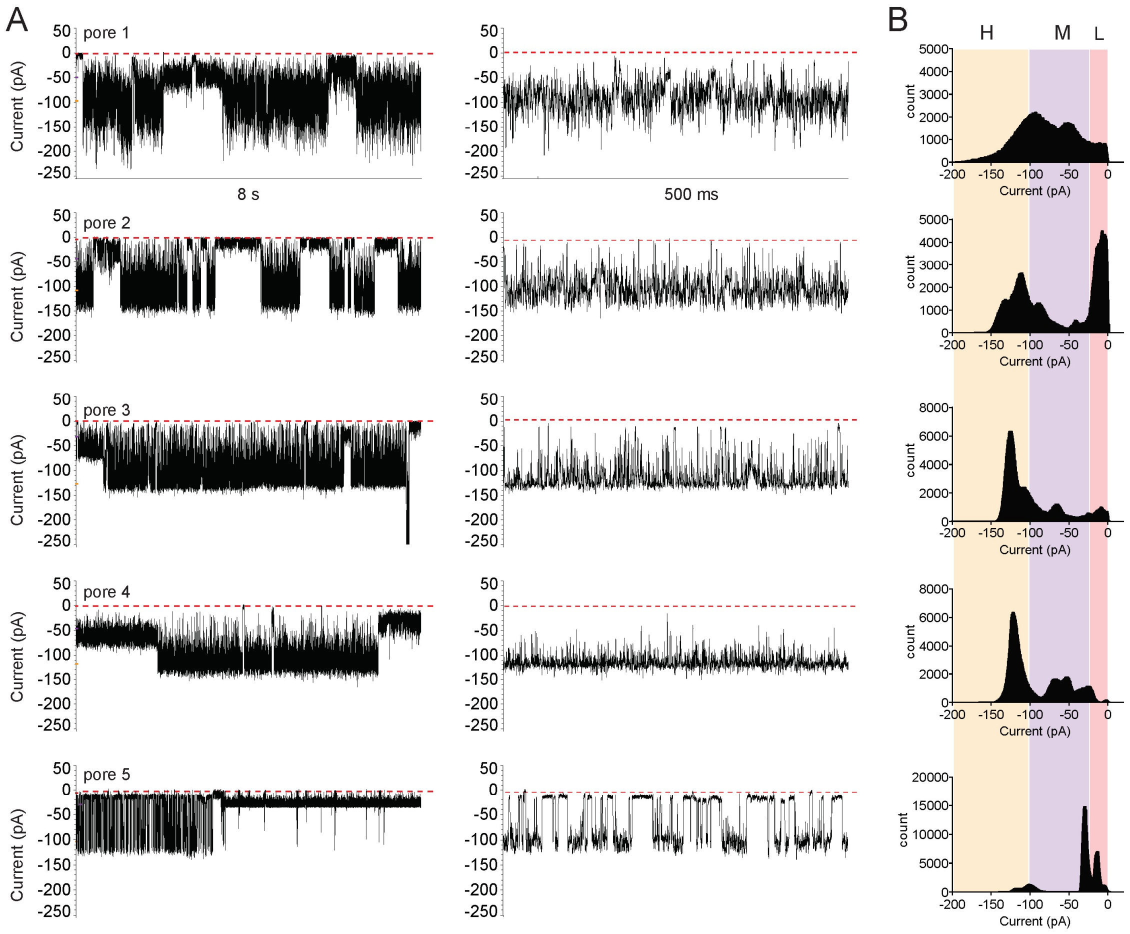

Supplemental Figure 17. **Single-channel conductivity recordings of the wild-type PelB.**

(**A**) Filtered traces recorded on individual $PelB_{WT}$ molecules show fluctuations in the ion currents. Next to each 8-s trace, a zoom-in of 500 ms is shown.

(**B**) Distributions of the ion currents plotted as histograms based on all-points 8-s traces from the panel A. The regions of low (L), median (M) and high (H) conductivity are highlighted, as described in the main text.

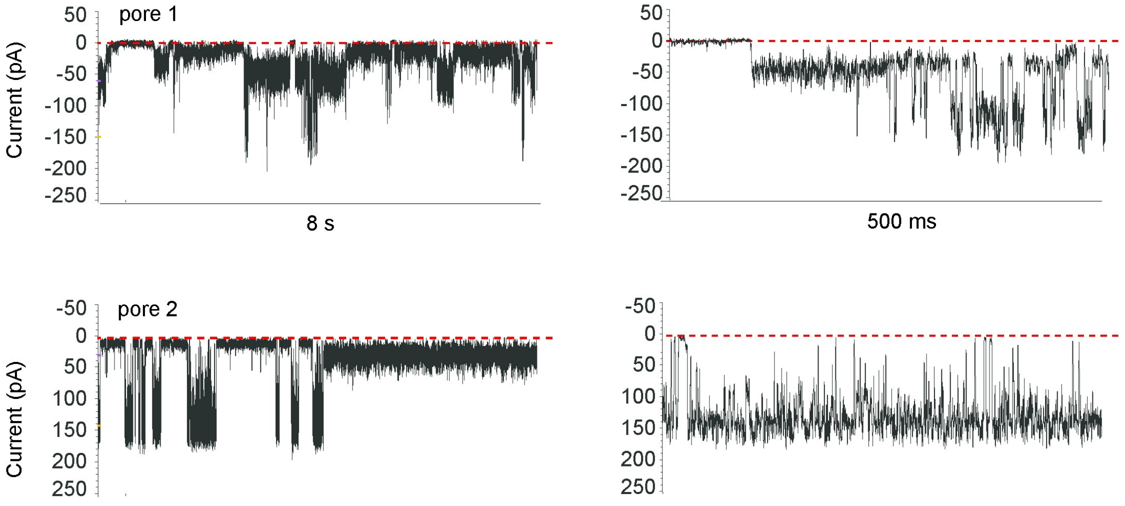

Supplemental Figure 18. Single-channel recordings of the PelB-TPR variant. Filtered traces recorded on individual PelB-TPR molecules show fluctuations in the ion currents. Next to each 8-s trace, a zoom-in of 500 ms is shown.

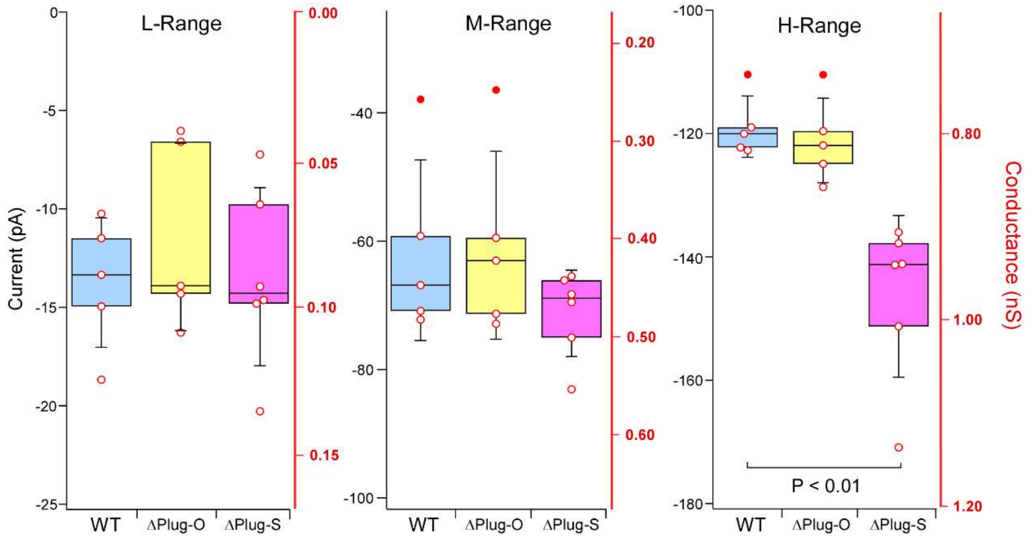

Supplemental Figure 19. Conductivity of the measured PelB variants. The average current value within each range (L, M and H) and the corresponding conductance measured at the single-molecule level are shown for $PelB_{WT}$, (blue), PelB ΔPlug-O (yellow) and PelB ΔPlug-S (violet). Each marker indicates a measurement of an individual PelB molecule (n=5 for $PelB_{WT}$ and PelB ΔPlug-O, n=6 for PelB ΔPlug-S). The plots show the median values, the 25th and 75th percentile (boxes) and the standard deviations (whiskers). The outliers (defined by Tukey) are shown as filled markers. The statistically significant difference found for the PelB ΔPlug-S current in the high- conductivity range (H-range) is indicated (p = 0.0017; one-way ANOVA test).

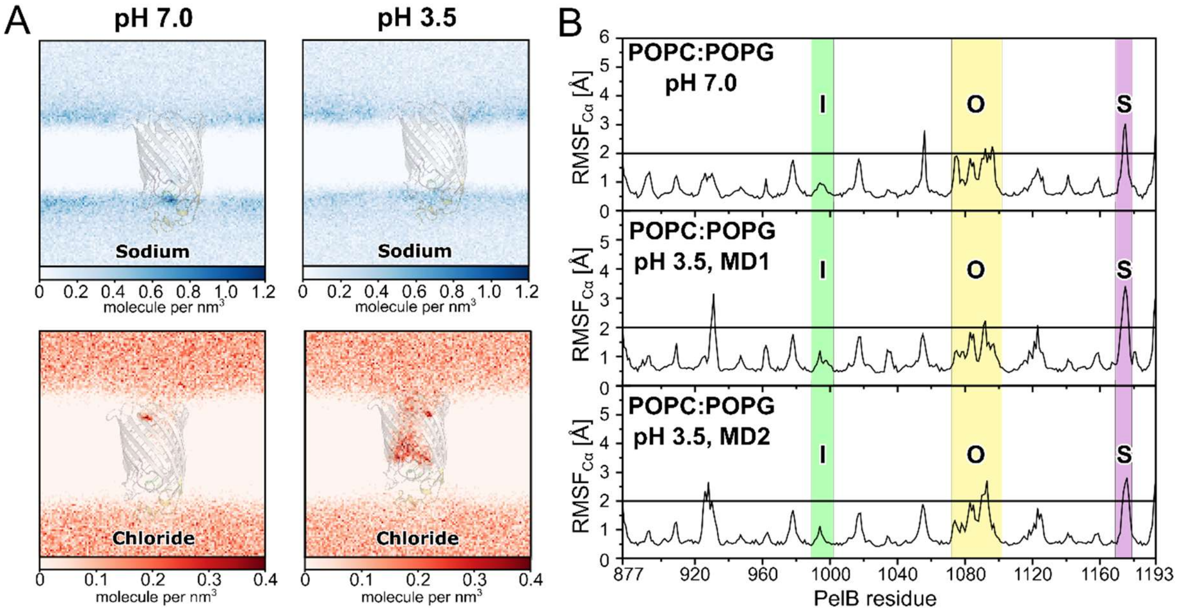

Supplemental Figure 20. Acidic pH affects the ion distribution, but not the conformational dynamics of PelB.

(A) Comparison of sodium (top) and chloride (bottom) distribution density maps across the PelB β-barrel at pH 7.0 vs. pH 3.5. The chloride anions enter the protonated barrel at the acidic conditions.

(B) The conformational flexibility of PelB in the symmetric POPC:POPG lipid bilayers at pH 7.0 vs pH 3.5.

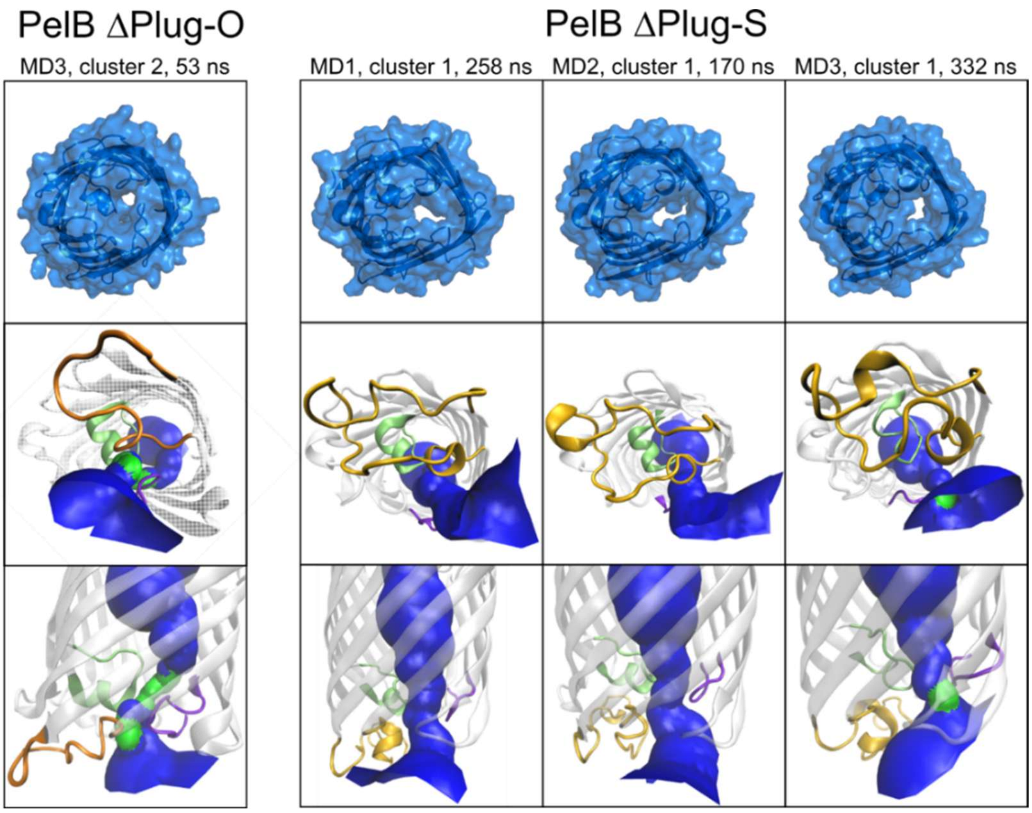

Supplemental Figure 21. The pore analysis of PelB ΔPlug-O and ΔPlug-S variants.

Top raw: Surface representation of the selected conformations, manifesting a tunnel in the PelB variants. The tunnel color-coding is according to Suppl. Figure 15.

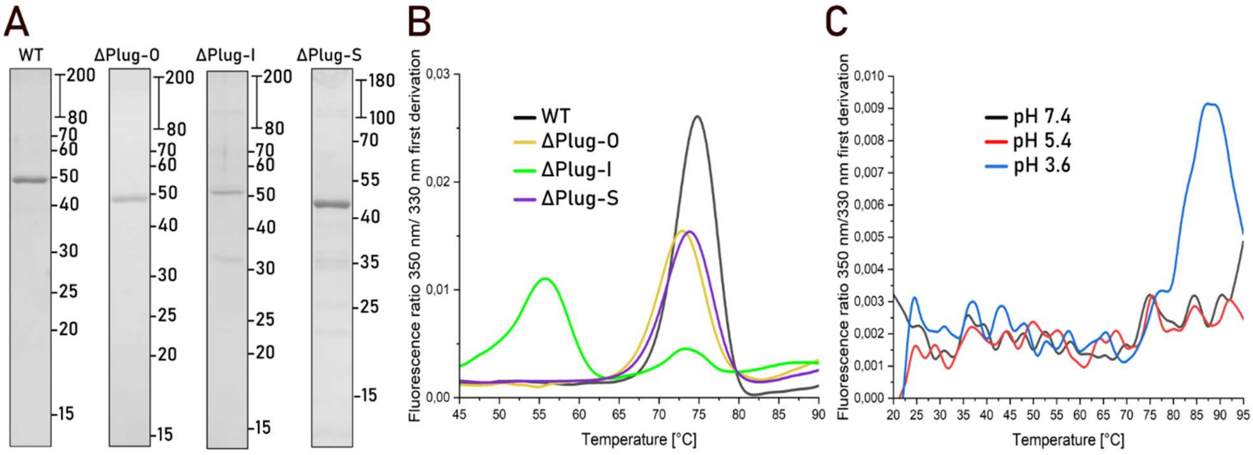

Supplemental Figure 22. **Thermal stability of the PelB variants.**

(**A**) SDS-PAGE of the PelB variants in DDM purified via IMAC and SEC.

(**B**) Thermal denaturation of the detergent-solubilized PelB variants monitored through changes in the intrinsic tryptophan fluorescence by nanoDSF.

(**C**) No thermal denaturation of the liposome-embedded wild-type PelB is resolved below 95 °C at pH 7.5 and pH 5.5. For pH 3.5, the denaturation occurs between 85 and 90 °C.

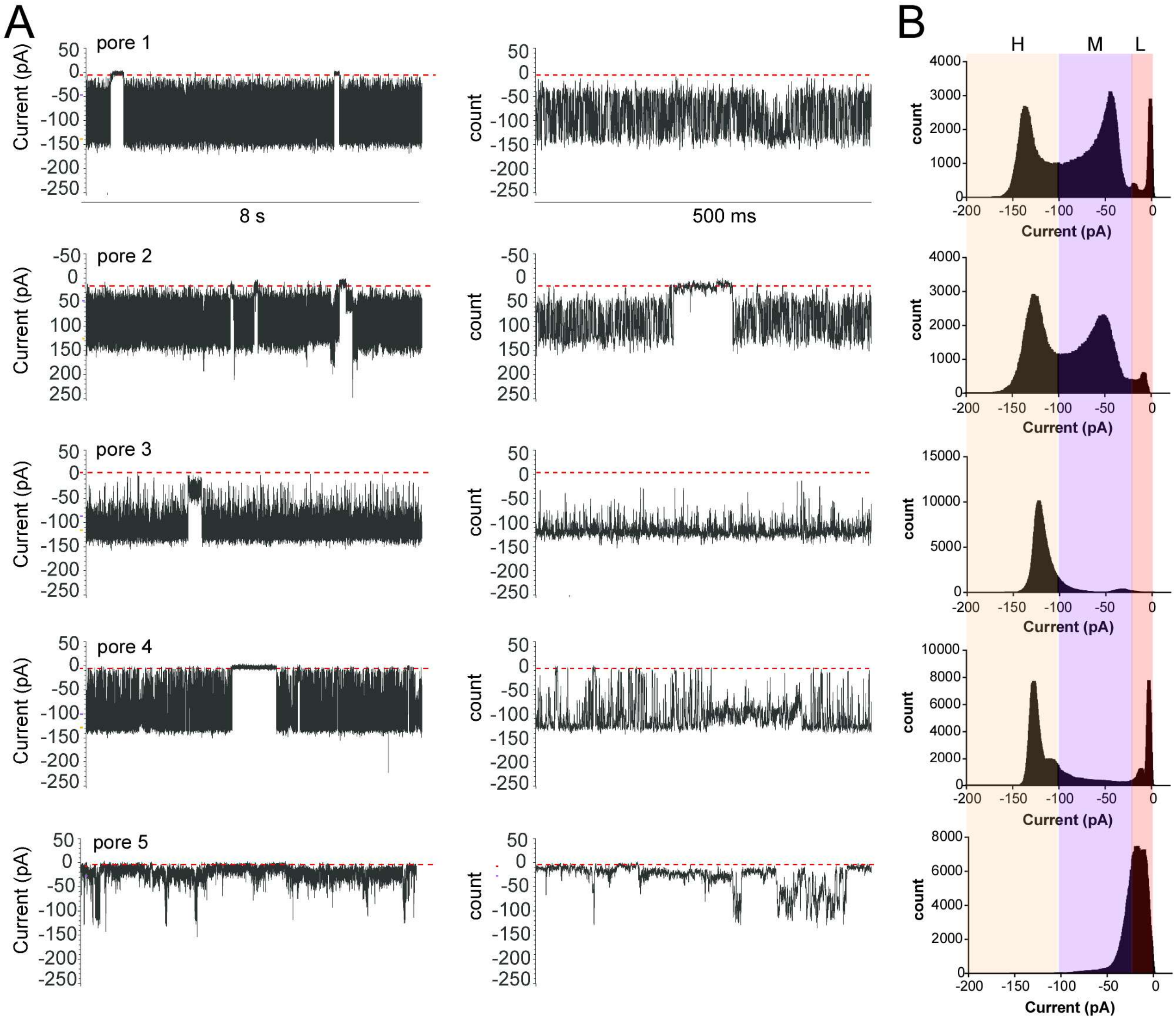

Supplemental Figure 23. Single-channel recordings of PelB ∆Plug-O.

(**A**) Filtered traces recorded on individual PelB ∆Plug-O molecules show fluctuations in the ion currents. Next to each 8-s trace, a zoom-in of 500 ms is shown.

(**B**) Distributions of the ion currents plotted as histograms based on all-points 8-s traces from the panel A. The regions of low (L), median (M) and high (H) conductivity are highlighted, as described in the main text.

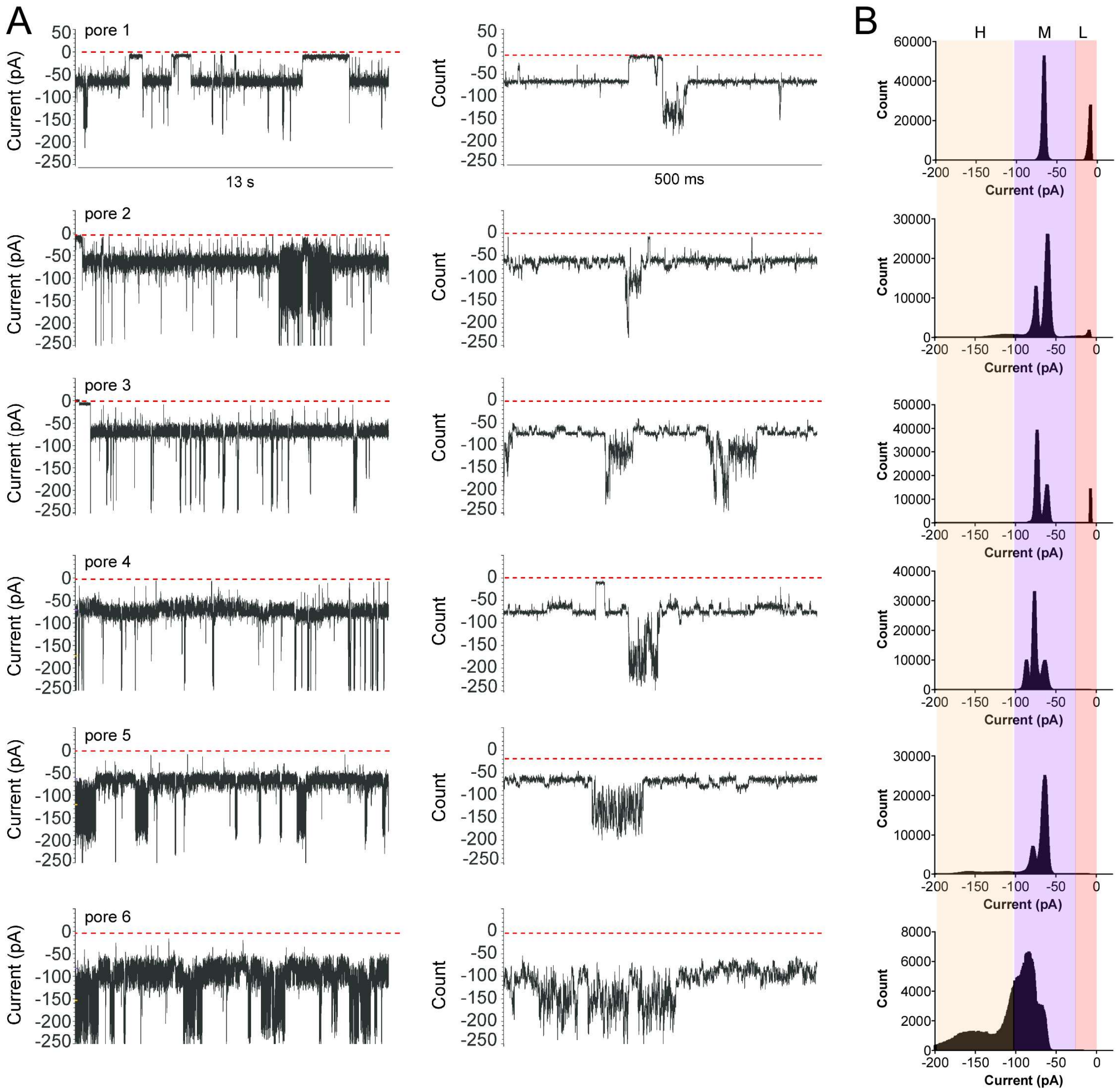

Supplemental Figure 24. Single-channel recordings of PelB ΔPlug-S.

(**A**) Filtered traces recorded on individual PelB ΔPlug-S molecules show fluctuations in the ion currents. Next to each 13-s trace, a zoom-in of 500 ms is shown.

(**B**) Distributions of the ion currents plotted as histograms based on all-points 13-s traces from the panel A. The regions of low (L), median (M) and high (H) conductivity are highlighted, as described in the main text.

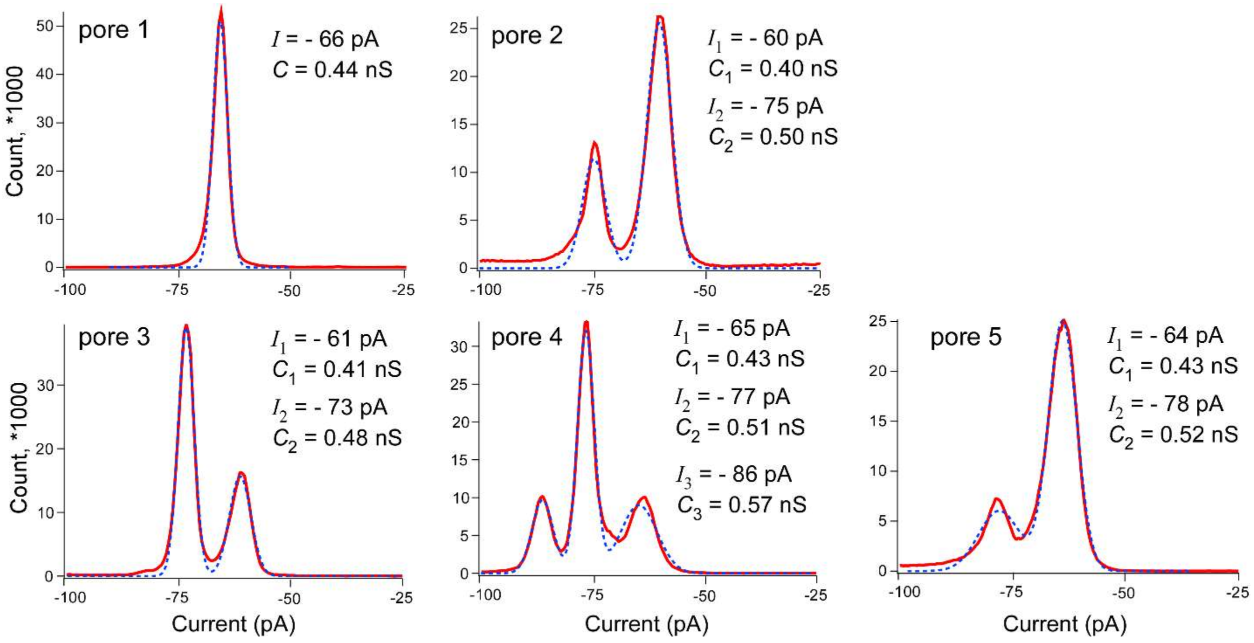

Supplemental Figure 25. Conductance of the sub-states resolved for PelB ∆Plug-S.

Measured ion currents and the calculated conductance values of the individual sub-states, as determined from Gaussian fitting (dashed blue lines) of the histograms within the M-range from Suppl. Figure 24 (solid red lines).

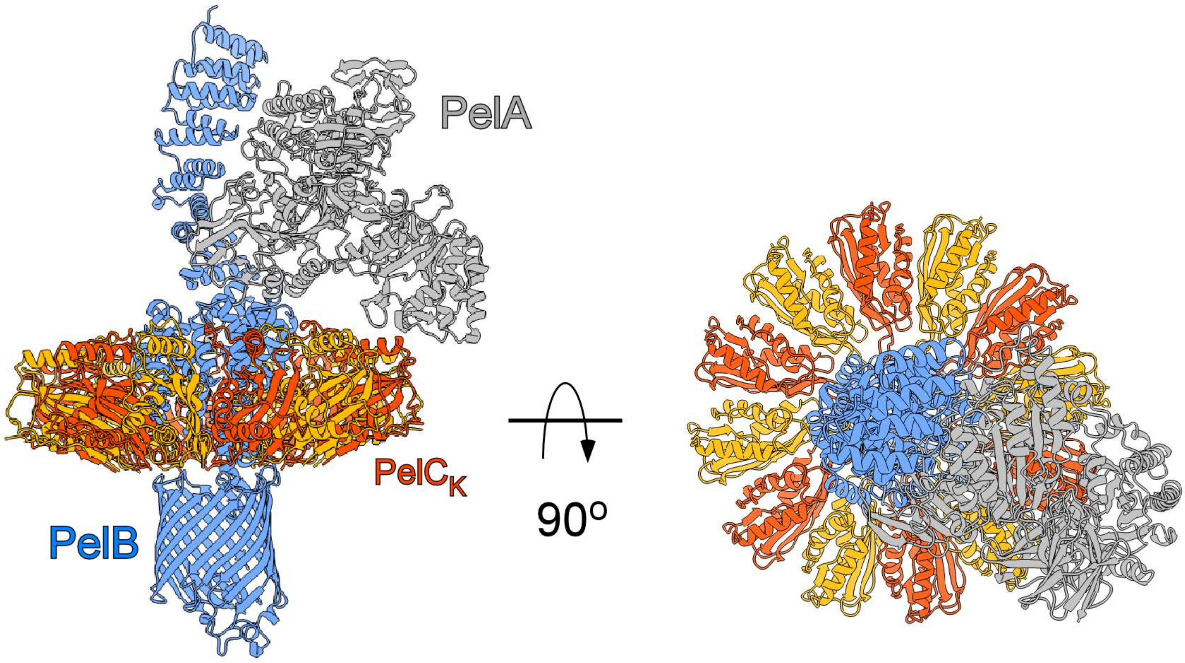

Supplemental Figure 26. AlphaFold3-based model of the putative PelA-PelBC complex. The hydrolase domain of PelB-bound PelA reaches PelC subunit K. PelB model is shown from the residue 318.
